# Three Years with the Army of the Potomac—A Personal Military History

**Published:** 1912-02

**Authors:** William Warren Potter

**Affiliations:** Buffalo, N. Y., Brevet Lieutenant Colonel, U. S. Volunteers; Surgeon in Charge of First Division Field Hospital Second Army Corps; Surgeon 57th Regiment New York Volunteers; Assistant Surgeon 49th Regiment New York Volunteers; Recorder Second Division Hospital Sixth Army Corps, etc., etc.


### Three Years with the Army of the Potomac—A Personal Military History

By William Warren Potter, M.D., Buffalo, N. Y., Brevet Lieutenant Colonel, U. S. Volunteers; Surgeon in Charge of First Division Field Hospital Second Army Corps; Surgeon 57th Regiment New York Volunteers; Assistant Surgeon 49th Regiment New York Volunteers; Recorder Second Division Hospital Sixth Army Corps, etc., etc.

April 27, 1863, 3 p. m. We are under orders to move at half an hour's notice, and all expect to be off before morning. We have now been in this camp over three months, and in the vicinity of Falmouth much longer; so it is desirable that a change should be made, even if we go but a short distance. The Fifth Corps is moving on our right today, and we received a call from Dr. Dean, who has been promoted to be surgeon of the 140th New York in that Corps. He is full of animation as usual, but only stayed with us a few minutes while his regiment was passing.

On the morning of the 28th, Tuesday, we marched at sunrise, and bivouacked for the night near Banks's ford. On the afternoon of the 29th we went forward to U. S. Ford, and bivouacked again in the rain and mud. A bridge was laid on the morning there during the greater part of the forenoon of May 1, and then of the 30th, and we crossed the Rappahannock the same afternoon, bivouacking near Chancellorsville late that night. We lay moved out beyond Chancellorsville about a mile on the road to Fredericksburg. The first division was acting in connection with the Fifth Corps at this time, and we reached the full view of the enemy's position, fully expecting to fight him. But about two oclock p.m., we received an order directing us to withdraw and fall back on Chancellorsville. We did this relucantly and slowly, finally forming near the Chancellor House in support of artillery. While I was sitting on my horse awaiting events, I had an opportunity to survey the position. The field was full of troops, artillery, ammunition wagons, and ambulances, and a few shells from the enemy, which we were expecting every moment, would have created a panic, I am sure. While thus waiting, the head of the Third Corps, with General Sickles and staff at its head, came up the U. S. Ford Road, and deployed on the right of Chancellorsville. Finally, we were given our position well out to the front about the center, and on one of the roads leading to Fredericksburg.

The First Division hospital was located in the woods about half a mile in rear of the Chancellor House, but we were not allowed to bring up our wagons, as nothing on wheels was permitted on this side of the river excepting artillery, ambulances, and ammunition wagons. So we had the men gather leaves, spread blankets upon them, and thus prepared beds for the wounded. Early Saturday morning I amputated a finger for a man who was wounded the night before, and soon the wounded began to arrive at our hospital. During the day Colonel Nelson A. Miles, 61st New York, was brought in wounded under the conduct of ambulance sergeant Joice, of the same regiment, and it was thought at first that his wound was mortal, as he was suffering from collapse from a bullet wound in the abdomen. He rallied, however, and the missile was cut out of his back near the spine, it having passed around between the muscular layers. He recovered promptly, and came back in time for Gettysburg. Saturday evening, just at dark, the 11th Corps gave way on our right, and their stragglers came through the woods, where our hospital was located, in such numbers as to nearly overrun us, and really endanger our wounded. The stampede was soon checked, however, though the woods were filled with stragglers most of the night. Sometime during the evening I saw Dr. Van Aernam, of the 154th New York, who told me his regiment was nearby. Near eleven o'clock P. M., I laid down on a blanket with A. B. Talcott, the Herald correspondent with the Second Corps, and while we were talking the artillery had a severe duel, the shells bursting above the tree-tops, and the explosions lighting up the heavens most brilliantly. This died away after midnight to an occasional gun, after which I obtained some sleep.

May 3. This Sunday morning we were up with the dawn, the fighting soon began and then the wounded poured in so we were busy, too much so to think about the ever shifting lines of battle, and to realize that a new line was being laid out which came very near our position. So the fighting went on during Sunday, to be renewed Monday morning, with disjointed and fitful intervals of quietude, only to be again broken by the sound of musketry and cannon, now in fearful proximity to our hospital. One of our wounded men had been re-wounded while lying on the ground, and one of the Ambulance Corps was killed while directing the unloading of an ambulance at the Hospital station. But all things must have an ending, and so with battles. On the afternoon of Monday, May 4, an ambulance train was loaded with wounded for shipment to Potomac Creek Hospital, which was located about half way between Falmouth and

Acquia Creek Landing, and I was ordered to take charge of it. We started about three o'clock P. M., crossed at U. S. Ford, and moved along the north bank of the Rappahannock until near Bank's Ford, then diverged to the left and struck across the country to Potomac Creek. It was near dark when we passed along the region of Bank's Ford, and we could plainly see the bursting of the shells where Sedgwick was having a struggle to cover the crossing of his Corps, the 6th, at this point. Finally, late at night I reached the Hospital and delivered the wounded, glad to get where I could have a night's sleep again, without fear of being awakened by the sound of guns and the noise of contending armies.

The next day, Tuesday, May 5, I was ordered on duty at the hospital, where I remained nearly three weeks, when I was relieved at my own request, as the wounded were all cared for as far as immediate operations were concerned. The surgical staff here is not adequate to do all the work, hence the Surgeon in charge was anxious to retain me. Our loss in the late battle has been heavy, and large numbers of the wounded have fallen into the hands of the enemy. I came near being hit myself several times, so near, indeed, that men on each side of me were killed and wounded. I am nearly worn out with fatigue and hunger, but shall soon recuperate here.

May 9. I have been busy daily since my arrival here, have made a number of operations, and shall amputate an arm the first thing tomorrow morning. Some estimate our loss at the Battle of Chancellorsville at 20,000 men, killed and wounded; but, at all events, we were ingloriously defeated—beaten in detail because our commander was not equal to the emergency.

May 13. Still at Potomac Creek Hospital and as busy as ever. We are now receiving the wounded that fell into the enemy's hands. They were delivered under flag of truce, and are glad enough to get with us. One man, whose wounds I dressed, gave me a Richmond paper of May 11, containing an account of the death of Stonewall Jackson, which I sent to my wife and it is still preserved with my papers.

May 15. Still busy with the wounded, and have three or four operations to make tomorrow morning. Until today the weather has been very hot, but a cooler wave has struck us which makes the wounded men more comfortable. As soon as the wounded are sent away I shall return to my regiment.

May 18. The weather is very hot, and it is very fatiguing to work over the wounded at such a time. Just at evening I rode over to the 6th Corps Hospital, where I saw Dr. Jenkins of the 49th, on duty. He says his regiment lost very few men in the late battle. The 57th lost two killed, and twenty-nine wounded, besides a few missing. I visited the 136th regiment on Saturday, the 16th, where I saw Gad Parker and others who were all right.

May 20. The weather is still hot and I am suffering with the toothache, which adds very materially to my discomforts. The wounded are doing well for the most part, and I shall probably get away very soon. Dr. Rowland, of the 61st, New York, is associated with me here, and is a very competent medical officer.

Sunday, May 24. I am once more in camp with my regiment, and am very glad to get back again. I was relieved from duty with the hospital yesterday, and started for the 57th at once. All the operations had been made and I could not derive any benefit from a longer stay there, so I applied to be relieved. The weather is so hot I am not anxious to work any harder than is absolutely necessary, and here with the regiment I have very little to do, as we have but two men on the sick list.

May 26. The 57th is in very nearly the same place where I wintered, having returned to the old camp after the battle. It seemed like getting home again to the boys. I expect to go to Acquia Creek Landing today to make arrangements for ice for the hospitals of the Brigade, similar to the plan of last year at Harrison's Landing.

May 28. Very hot. Our Division has just been reviewed by General Hancock, who now commands the 2d Corps. Rumor has it that General Couch, after Chancellorsville, refused to serve longer under General Hooker, and was relieved at his own request. The 21st regiment has been mustered out of service on expiration of term, and is now home in Buffalo where it has been received with honor. It was a good regiment and deserves recognition at the hands of the citizens of Buffalo, for the service it has so honorably performed.

May 30. Everything is extremely dull in and about the Army these days, though day before yesterday, the 28th, we had orders to be ready to fall in at a moment's notice; but it created no excitement as everybody regarded it as a bit of a scare. It is rumored that Lee contemplates an attack upon our right, but I hardly think it probable that he will. The whole Army is very anxious for him to do so, for we think we could whip him handsomely if he would only attack us once. We have always been the attacking party, and that works to our disadvantage. I made a large number of operations after Chancellorsville, and they all did well with one exception. This case—a man belonging to the 88th New York, Irish Brigade—I amputated midway between the ankle and knee joints. Eight days afterwards his nurse, in removing the dressings one morning, tore out all the ligatures causing alarming hemorrhage. This we arrested as soon as possible, but as he had two other wounds, the discharge from them together with the loss of blood proved too exhausting, and he died at the end of ten days. The leaves of absence for ten days that have been granted for the past few months have now been suspended, and officers can only get away at present on Surgeons' certificate of disability, or for five days in cases of great urgency. A great many officers are, as a consequence, obtaining sick leaves just now.

Monday, June 1. Yesterday Major Bell and I dined with Dr. Hewett, Surgeon of the 119th New York. The doctor is a friend of Major Bell's, and we went by special invitation at six o'clock P. M., at which hour, with several other guests, we sat down to the table. The menu consisted of oyster soup, claret punch, spring lamb and mint sauce, potatoes, green peas, asparagus, etc.; apple pie, ice cream, fruit and coffee. The company did not break up until near midnight, and Major Bell and I stayed all night at Dr. Hewett's. On our way home this morning we called at General Hooker's headquarters on some friends.

June 4. Yesterday I paid a visit to the 49th, and had a nice old-fashioned visit with Dr. Hall and others. General Howe gave a ball there last night, but I did not remain. Mrs. Colonel Lewis is visiting her husband in the Vermont Brigade. The Lewis's are from Buffalo.

June 6. The rebels are reported as having left their fortifications above Fredericksburg, and moving we know not whither. Heavy firing has been heard below the town this P. M., but it was chiefly to protect the Bridge Building. Two pontoons are across at Franklin's old crossing, and the Sixth Corps has thrown over a division, I presume to feel the enemy and see if he is still there in force. We are under orders to be ready to move at a moment's notice with three days' rations and sixty rounds of ammunition, which looks as though something was up. Bands are playing in all directions tonight, eleven-thirty o'clock, an ominous sign of a move.

June 8. We are still in the same old camp, but are constantly receiving orders indicative of a movement, and today all our sick were sent away—a pretty sure indication of activity. Yesterday I visited the left of the Army to see what the Sixth Corps was about, and found the "Old Division," the 2d, across the Rappahannock, the 49th on the extreme left of the line near the Bernard House, where our hospital was at the first battle of Fredericksburg in December last. The House itself is a partial ruin from fire, though being of stone the walls yet stand intact. (See illustration in the "Century," August, 1886, Page 637.) I spent about an hour there, and had a pleasant visit with Major Ellis.

Recrossing the river at five o'clock P. M., I called at the Division Hospital where I found Dr. Hall in charge. His wife had arrived about an hour before, and he was very happy. I met Mrs. Colonel Lewis across the river, whither she had gone to pay her husband a visit. She was in good spirits and quite brave, having had an opportunity to see the Division in line of battle.

June 11. We have not moved yet, but are expecting orders at any minute. I visited the Sixth Corps again yesterday, where I saw Dr. Hall and wife, Colonel Bidwell, Major Ellis, and others. The 49th has been relieved from duty on the other side of the river, and is now encamped on the heights on this side. A great number of ladies are visiting the Sixth Corps now. Day before yesterday the Paymaster made his appearance, and I drew pay for the balance of January, and for February, March and April, sending $300.00 home. No military commission was ever convened in my case, so I drew pay upon the ordinary vouchers, filing my muster-in roll as Surgeon as well.

### THE GETTYSBURG CAMPAIGN.

June 13, 7.30 P. M. I am strongly of the opinion that we shall march before tomorrow at this time, but we are, of course, in ignorance of our destination. Just now the Quartermaster informs me that he has orders to take the Corps supply train to Stafford C. H. tonight, with the expectation that we follow early tomorrow morning.

June 17. Near Fairfax Station on the Orange & Alexandra R. R. we marched on Sunday, the 14th, and reached this place last night, coming via Dumfries. The First Division has had some slight skirmishing with the enemy, but no general engagement as yet. We are now about sixteen miles from Alexandria. The North must be in a state of excitement now, for the rebels are reported to have invaded Pennsylvania. I am glad they are there, for it will undoubtedly have a tendency to shorten the war.

June 18. We have moved two miles to Sangster's Station since yesterday, and are now about eighteen miles from Alexandria. The day has been very hot, but now, four o'clock P. M., there are signs of rain. Major Bell leaves tonight for Washington to enter the veteran Reserve Corps, and has been ordered to report for duty to Major Diven at Elmira.

June 19. It rained hard all night, and fortunately we did not move. The 49th is only about a mile from us, and Colonel

Bidwell gave me his photograph when I was there day before yesterday. Dr. Hall's wife has gone home, but Mrs. Lewis is still with her husband.

June 22. Near Gainesville. We camped at Centerville Friday night, and Saturday moved up here where we have been since. We are six miles from Thoroughfare Gap. Yesterday Pleasanton's Cavalry and a part of the Fifth Corps had a battle with Stuart the other side of the Bull Run Mountains, near White Plains where we lay last fall when McClellan was removed. The rumor is that Stuart was beaten. Last night General Stahl, with several thousand cavalry, passed along the road towards Warrenton. We are on the Warrenton Turnpike about twelve miles from Warrenton, in the most beautiful part of Virginia that I have yet seen. Our camp is located in a delightful meadow, and a cool breeze is rendering the air quite balmy. It looks, however, as though we should move today.

June 28. Near Frederick, Md. We left Gainesville Thursday noon, the 25th, crossed the Potomac at Edward's Ferry on the 26th, and reached Frederick at noon today. I don't know the distance from Gainesville here, but we have marched day and night since leaving there, with only short intervals of rest. General Hancock passed the column about seven o'clock this morning, and I rode with him a short distance, having a pleasant visit with himself and staff. It is said here this evening that General Hooker has been relieved from the command of the Army, and that General Meade, of the 5th Corps, has been appointed in his stead. We shall probably march early in the morning.

June 29. Last night at nine o'clock the 57th, with a section of artillery, was ordered to guard a bridge over the Monocacy River about two miles north of Frederick, and we remained out upon this duty all night. It seems there was a suspicion that the enemy might undertake to burn the bridge, but if any such idea ever pervaded his mind he made no attempt to carry it out while we were there. This, Monday, morning we were ordered to join the corps which was on the march towards Westminster; but owing to some delay in the transmission of the order we did not start until near nine o'clock, and consequently did not overtake the command until after midnight. I don't know just what time we did make junction with the remainder of our Division for, sometime during the night, being tired and hungry I reined into a lot, and sat down on a cock of hay with Captain Rose, our Commissary of Subsistence. Discovering a cow in the field near by, for it was moonlight, we got one of the soldiers to milk her and, after refreshing ourselves with hardtack and milk, we soon fell asleep. When I awoke it was daylight, my horse had straggled off a little way, and was grazing in a clover field. I was alone, Captain Rose had gone, and I could see no troops anywhere; so I mounted my horse and rode on. I soon came across the regiment, and then stopped at a farm house where I got breakfast, and fed my horse some grain purchased of a farmer. At this house I wrote a letter home at five o'clock A. M., which I mailed at Union Bridge, Md., a small village near by, which was, however, called Uniontown in our orders. We remained all day, the 30th, resting near Uniontown, and were there mustered for pay in the afternoon.

(Continued.)

